# A DEK domain-containing protein GhDEK2D mediated *Gossypium hirsutum* enhanced resistance to *Verticillium dahliae*

**DOI:** 10.1080/15592324.2021.2024738

**Published:** 2022-01-16

**Authors:** Jinglong Zhou, Lihong Zhao, Yajie Wu, Xiaojian Zhang, Sheng Cheng, Feng Wei, Yalin Zhang, Heqin Zhu, Yi Zhou, Zili Feng, Hongjie Feng

**Affiliations:** a College of Agriculture, Yangtze University, Jingzhou, China; b State Key Laboratory of Cotton Biology, Institute of Cotton Research of Chinese Academy of Agricultural Sciences, Anyang, China; c Zhengzhou Research Base, State Key Laboratory of Cotton Biology, Zhengzhou University, Zhengzhou, China

**Keywords:** *Verticillium dahliae* (*Vd*), Dek domain containing protein, cotton resistance

## Abstract

DEK is associated with DNA replication and break repair, mRNA splicing, and transcriptional regulation, which had been studied in humans and mammals. The function of DEK in plants was poorly understood. In this study, *GhDEK*2D was identified in *Gossypium hirsutum* by genome-wide and post-translational modifications. GhDEK2D had been phosphorylated, acetylated and ubiquitylated under *Verticillium dahliae* (*Vd*) challenge. The *GhDEK2D*-silenced cotton decreased resistance against *Vd*. In *GhDEK2D*-silenced cotton plants, the reactive oxygen species was activated, the callose, xylogen, hypersensitive reaction (HR) and expression levels of defense-related genes were reduced. Homozygous overexpressing-GhDEK2D transgenic *Arabidopsis* lines were more resistant to Verticillium wilt (Vw). We propose that GhDEK2D was a potential molecular target for improving resistance to Vw in cotton.

## 1. Introduction

Cotton is one of the most important economic,^1^ textile fiber, and oilseed crop.^2^ *G. hirsutum* is characterized by high yield and quality fiber, and becomes the most cultivated species in the world cotton production.^3^ However, cotton production was affected by abiotic and biotic stresses in many regions of the world, among them, Vw is a destructive biotic stresses, which caused by the soil-borne fungus *Vd*.^4,5^ For the past few years, in the wake of climatic change, long-term mono-cropping and frequent introduction of a fine variety led to break out Vw in cotton.^6^ The fungus can form dormant structures called microsclerotia that can survive in the soil for years without a host and infect subsequent crops, so that Vw is difficult to control in cotton.

Post-translational modifications (PTMs) play a role in every aspect of cell biology by regulating many cellular processes.^7^ Phosphorylation, acetylation, and ubiquitination are important PTMs. They are necessary for functional signal transduction and metabolism, and are involved in chromatin conformation regulation, transcription, and cell metabolism.^8^ PTMs also affects cell localization, stability, interaction, and enzyme activity.^9^ Activity of proteins Phosphorylation, one of the most extensive modifications of proteins, is a reversible process catalyzed by a kinase that transfers a phosphorylating group in ATP to the hydroxyl group of a specific serine, threonine or tyrosine residue in the target protein.^10,11^ Phosphorylation is a key process that regulates plant growth, development and stress response.^12^ Reversible phosphorylation of serine, threonine, and tyrosine residues performs many important functions, such as altering protein conformation and activation states, protein stability and degradation, subcellular localization, and interaction with protein substrates.^13^ Mining resistance genes has become the major way to sustain cotton industry. Protein post-translational modifications is major defense mechanism for regulating plant immune.^14^ A growing body of PTMs profiling has provide convenience to excavate new resources.

DEK was originally cloned from acute myeloid leukemia and is a bona fide oncoprotein associated with many different tumor types, as well as with stem cell and progenitor cell quality.^15–17^ DEK was found in all multicellular organisms.^18^ Chromatin structure plays an important role in various processes.^19^ DEK proteins are involved in the control of various chromatin related processes.^20^ The function of DEK has been widely studied in mammals, and it takes part in DNA replication and repair, mRNA splicing, transcription regulation, cell lifetime, division and differentiation.^21–24^ In *Arabidopsis*, there are four DEK proteins, and named DEK1, DEK2, DEK3 and DEK4. DEK has been reported to be present in the nucleolus. DEK3 affects nucleosome occupancy and chromatin accessibility, and adjusts the expression levels of its target genes.^25^ The correct expression of DEK3 can regulate the response of plants to stress conditions such as high salt and heat shock. *Arabidopsis* DEK3 and DEK4 prevent early flowering by activating transcription of key flowering inhibitors.^26^ Silencing DEK2 or DEK4 in tomatoes accumulates more ROS during pathogen infection and alters the expression of *PR1b, PR2, LaPA* and *PIN2*. The results showed that DEK is involved in the regulation of defense response to pathogens.^27^

In this study, there were six DEKs, which were highly conservative proteins in *G. hirsutum*. GhDEK2D had been phosphorylated, acetylated and ubiquitylated after *Vd* inoculation. The *GhDEK2D*-silenced cottons decreased resistance against *Vd*. In contrast, overexpressing-GhDEK2D enhanced *Arabidopsis* resistance to *Vd*. In *GhDEK2D*-silenced cotton plants, ROS, callose, xylogen, expression levels of defense-related genes and HR genes were reduced. These all indicate that GhDEK2D involves in defense response’ regulation to pathogen in *G. hirsutum*.

## 2. Materials and methods

### 2.1 Fungal strain, plant lines, and culture conditions

In this study, a virulent, defoliating *Vd* Vd080 was used to infect plants,^28^ and its cultural method referenced Zhou et al.^5^

A *G. hirsutum* cultivar Zhongzhimian NO. 2 is resistance to *Vd*. They were grown in a conditioned greenhouse with a 16 h/8 h photoperiod at 22°C to 28°C. *A. thaliana* (Columbia) was grown in a conditioned greenhouse with a 16 h/8 h photoperiod at 22°C. The culture method of *Nicotiana benthamiana* is the same as that of Zhongzhimian NO. 2.

### 2.2 Sequence retrieval and identification of DEK genes

Twenty *Arabidopsis* DEK protein sequences were used as queries to conduct a homologous blast search against *G. hirsutum, G. raimondii, G. arboretum, G. barbadense* (https://cottonfgd.org/sequenceserver/),^29–32^ *G. darwinii* (https://phytozome-next.jgi.doe.gov/info/Gdarwinii_v1_1),^33^ *G. tomentosum* (https://phytozome-next.jgi.doe.gov/info/Gtomentosum_v1_1),^34^ *G. mustelinum* (https://phytozome-next.jgi.doe.gov/info/Gmustelinum_v1_1),^33^ *Populus trichocarpa* (https://phytozome-next.jgi.doe.gov/info/Ptrichocarpa_v4_1),^35^ *Zea mays* (https://phytozome-next.jgi.doe.gov/info/ZmaysB84_v1_2),^36^ *Manihot esculenta* (https://phytozome-next.jgi.doe.gov/info/Mesculenta_v8_1),^37^ *Solanum lycopersicum* (https://phytozome-next.jgi.doe.gov/info/Slycopersicum_ITAG4_0),^38^ *S. tuberosum* (https://phytozome-next.jgi.doe.gov/info/Stuberosum_v6_1)^39^ and *Linum usitatissimum* (https://phytozome-next.jgi.doe.gov/info/Lusitatissimum_v1_0)^40^ protein databases. The molecular weights (kDa) andisoelectric points (pI) of DEK proteins (*G. hirsutum, G. raimondii, G. arboretum* and *G. barbadense*) were surveyed from Cotton Functional Genomics Database.^41^

### 2.3 Phylogenetic, gene structure, conserved domain analysis and chromosomal mapping

A total of 4 *Arabidopsis* DEK protein sequences were downloaded from TAIR (https://www.arabidopsis.org/). Multiple sequence alignments of all identified DEK from cotton, *P. trichocarpa, Z. mays, S. lycopersicum, S. tuberosum, L. usitatissimum* and *Arabidopsis* were performed in ClustalX 2.0. After conducting a model test, a maximum likelihood (ML) phylogenic tree was constructed with the best substitution model using MEGA X software.^42^

TBtools was extracted to draw the gene structure of *DEK* based on the whole-genome sequence and annotation datas.^43^ Conserved motifs of DEK proteins were identified using the Multiple Em for Motif Elicitation Version 5.4.1 (https://meme-suite.org/meme/tools/meme) with the default parameters.^44^ The conserved domains of DEK proteins were identified by Batch Web CD-Search Tool (https://www.ncbi.nlm.nih.gov/Structure/bwrpsb/bwrpsb.cgi).^45,46^ The figures of gene structure, conserved motifs and domains were drew by TBtools software.^47^

Chromosomal position information about cotton DEKs was obtained from the CottonFGD webpage. *GhDEK, GrDEK, GaDEK* and *GbDEK* genes synteny and collinearity were determined and analyzed by the MCScanX software.^48^ And the distribution of *DEK* genes were draw used TBtools.

### 2.4 Transcriptome analysis of GhDEK in tissues of cotton and responded to Vd

The expression patterns of *GhDEK*s were exhibited in tissues, TM-1 transcriptome data from (Accession codes, SRA: PRJNA490626, https://www.ncbi.nlm.nih.gov/bioproject/?term=PRJNA490626).^49^ The Zhongzhimian NO. 2 plants treated with *Vd* at 12 and 24 hpi, quantitative reverse transcription PCR (RT-qPCR) was used to determine *GhDEK*s expression levels.

### 2.5 Virus-induced gene silencing (VIGS) of GhDEK2D in cotton

VIGS were performed to inhibit expression of *GhDEK2D*, the experimentation as Feng et al.^50^

### 2.6 Arabidopsis thaliana transformation

In order to express *GhDEK2D* in *A. thaliana*, the primers *OE-GhDEK2D*-F and *OE-GhDEK2D*-R (Table S2) were used to clone the *GhDEK2D*. The *GhDEK2D* was inserted into the p*CAMBIA3300-eGFP* vector containing a *glufosinate* (*Basta*) resistance gene for overexpression studies. T_0_ – T_3_ transgenic seeds were sprayed with 0.01% Basta and PCR to select positive transformants. The high *GhDEK2D* expression T_4_ lines were used to study.

### 2.7 Plant disease resistance assay

Plants (*A. tumefaciens* and cotton) disease resistance assay as Zhou et al.^5^

### 2.8 Visualization and quantification of H_2_O_2_, lignification and callose staining

To visualize the accumulation of H_2_O_2_, cotton leaves were collected 48 h after Vd080 inoculation and incubated in DAB solution (1 mg/mL, pH 7.5) for 8 h. leaves were decolorized in 95% ethanol until the green color was completely removed in boiling water bath. ROS accumulation of leaves in 70% glycerol was observed under a microscope.

Cotton lignification was examined with phloroglucinol staining. The experimental procedure referenced Zhou et al.^5^ Thickness measurement were manipulated using ImageJ software.

Cotton callose deposition was visualized by aniline blue staining. The experimental procedure referenced Zhou et al.^5^ Thickness measurement were manipulated using ImageJ software.

### 2.9 Expression of defense-related genes by RT-qPCR

Cotton leaves were excised 0, 3, 6, 9, 12, 24, and 48 h post inoculation for RT-qPCR. The RNA was extracted by RNAprep Pure Plant Kit (TIANGEN, Beijing, China). The cDNA was synthesized by TransScript® All‐in‐One first‐strand cDNA Synthesis Super Mix for qPCR reverse transcription kit (TransGen, Beijing, China). qPCR was execute by TransStart® Tip Green qPCR Super Mix kit (TransGen, Beijing, China). The specific primers of cotton defense-related genes were listed in Table S4. Technical replicates of three independent biological samples were performed.

### 2.10 Subcellular localization

The *A. tumefaciens* strain GV3101 containing p*CAMBIA3300-GhDEK2D-eGFP* vector was used for subcellular localization. The experimentation as Feng et al.^50^ DAPI was used to mark cell nucleus.

## 3. Results

### 3.1 Identification and phylogenetic analysis of DEK genes family in cotton

To identify putative *DEK* family genes in cotton, 4 *Arabidopsis* DEK protein sequences were used as queries, and then conducted a homologous blast search against *G. hirsutum* (CRI), *G. raimondii* (JGI), *G. arboretum* (CRI) and *G. barbadense* (CRI) protein databases (https://cottonfgd.org/sequenceserver/). The potential DEK sequences were further inspected whether containing the conserved DEK motif by the InterProScan databases (https://www.ebi.ac.uk/interpro/search/sequence/). We obtained 6, 2, 3, and 7 DEKs in *G. hirsutum, G. raimondii, G. arboretum* and *G. barbadense*, respectively. The lengths of DEK proteins ranged from 360 to 642 amino acids, the molecular weight (MW) ranged from 41.333 kDa to 73.261 kDa, and the isoelectric point (IP) ranged from 4.571 to 8.033 (Table S1).

**Figure 1.**
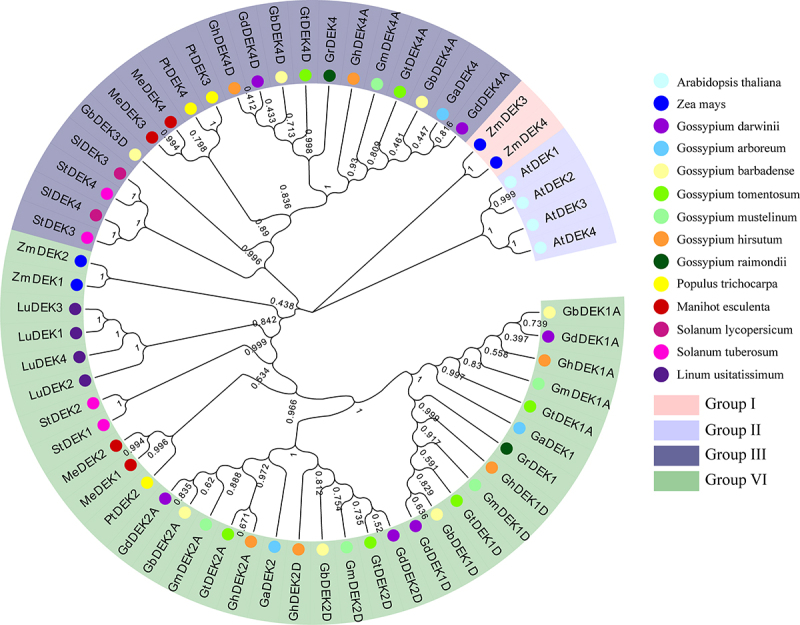
Phylogenetic analysis of DEK in cotton and *Arabidopsis.*

To further explore the evolutionary relationship of DEKs, 6 GhDEKs, 2 GrDEKs, 3 GaDEKs, 7 GbDEK, 6 GdDEK, 6 GtDEK, 5 GmDEK from cotton, 3 PtDEK from *P. trichocarpa*, 4 ZmDEK from *Z. mays*, 4 MeDEK from *M. esculenta*, 2 SlDEK from *S. lycopersicum*, 4 StDEK from *S. tuberosum*, 4 LuDEK from *L. usitatissimum* and 4 AtDEKs from *Arabidopsis* were used for constructing a phylogenetic tree by MEGA X software using the maximum likelihood (ML) method (Figure 1). The DEK family can be divided into four groups (I to IV), Group I only contained AtDEKs, Group II only contained 2 ZmDEKs, Group III contained 20 DEKs of 11 species, Group IV contained 30 DEKs of 12 species. The results suggested *DEK*s were highly conservative in respective species. The DEKs ID and names were listed in Table S2.

A phylogenetic tree of DEK proteins from *G. arboreum, G. raimondii, G. hirsutum, G. barbadense, G. darwinii, G. tomentosum, G. mustelinum, Populus trichocarpa, Zea mays, Manihot esculenta, Solanum lycopersicum, S. tuberosum, Linum usitatissimum* and *A. thaliana*. The full-length amino acid sequences of the DEK proteins were aligned using ClustalX in MEGA X. The unrooted tree was generated by the maximum likelihood (ML) method (n = 1000 bootstraps). Clades are distinguished by different-colored line.

### 3.2 Chromosomal location and gene synteny analysis of DEK genes in Gossypium

**Figure 2.**
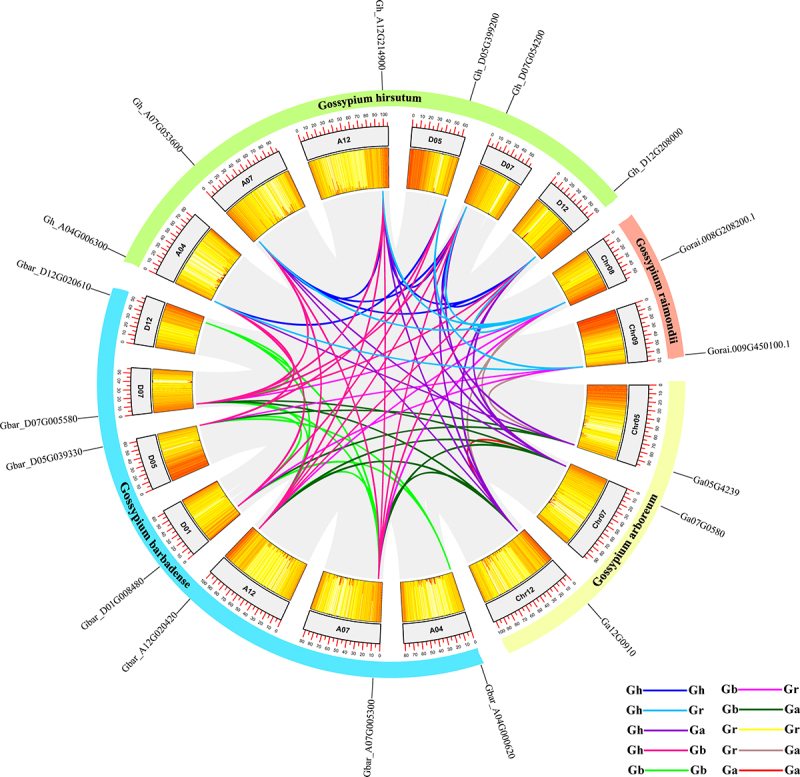
The synteny relationship of *DEK* genes in *G. arboreum, G. raimondii, G. hirsutum* and *G. barbadense.*

The chromosomal locations of genes were investigated by the complete *Gossypium* genome sequence. A total of six *GhDEK* genes were distributed throughout the Gh_A04, Gh_A07, Gh_A12, Gh_D05, Gh_D07, and Gh_D12 subgenome. Three *GaDEK*s were distributed throughout the Ga_Chr05, Ga_Chr07, and Ga_Chr12 subgenome. Two *GrDEK*s were distributed throughout the Gr_Chr08 and Gr_Chr09 subgenome. Seven *GbDEK*s were distributed throughout the Gb_A04, Gb_A07, Gb_A12, Gb_D01, Gb_D05, Gb_D07 and Gb_D12 subgenome (Figure 2).

MCScanX was used to identify genome collinearity and tandem repeats under default parameters, and TBtools was used to show collinearity. The different-colored lines represents paralogous gene pairs.

Syntenic analysis were performed to studies the relationships of *DEK* genes among the *G. hirsutum, G. raimondii, G. arboretum*, and *G. barbadense* (Figure 2). Among the *DEK* genes, six *GhDEK*s were the orthologous genes of the two *GrDEK*s, five *GbDEK*s and three *GaDEK*s, respectively. Five *GbDEK*s were the orthologous genes of the two *GrDEK*s and three *GaDEK*s, respectively. Two *GrDEK*s were the orthologous genes of the three *GaDEK*s. Some *DEK*s had not only one orthologous gene, such as *GrDEK1* had four genes (*GhDEK1A, GhDEK1D, GhDEK2A* and *GhDEK2D*) in *G. hirsutum*, three genes (*GbDEK1A, GbDEK2A* and *GbDEK2D*) in *G. barbadense*, and two genes (*GaDEK2* and *GaDEK1*) in *G. arboretum. GrDEK4* had two genes (*GhDEK4A* and *GhDEK4D*) in *G. hirsutum*, two genes (*GbDEK3D*, and *GbDEK4D*) in *G. barbadense*. More orthologous genes were displayed in Table S5. In addition, there were two paralogous gene pairs (*GhDEK1A*/*2A*/*1D*/*2D* and *GhDEK4A*/*4D*) in *G. hirsutum*, one paralogous gene pair (*GaDEK1*/*2*) in *G. arboretum*, two paralogous gene pairs (*GhDEK1A*/*2A*/*1D*/*2D* and *GhDEK3D*/*4A*/*4D*) in *G. barbadense*, and zero paralogous gene pair in *G. raimondii* genome (Table S3). Furthermore, all cotton DEKs were classified into WGD or segmental duplications (Table S3). In the expansion of the *DEK* gene family, WGD or segmental duplication might play a crucial role.

### 3.3 Gene structure, conserved motifs and domain of DEKs

**Figure 3.**
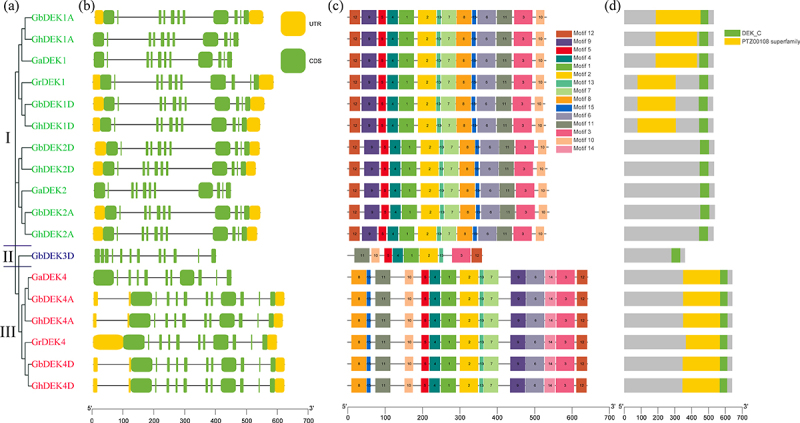
Homologous relationship, Structural, motif and domain analysis of DEKs in *G. arboreum, G. raimondii, G. hirsutum* and *G. barbadense.*

To better reveal the evolutionary relationships of the *DEK* gene family, a separate unrooted phylogenetic tree was constructed with *DEK* DNA sequences (Figure 3a). The comparative analysis of intron–exon structure showed that *GhDEK* gene length varied significantly. *DEK* genes had a highly rich distribution of introns in the regions, except GbDEK3D possessed 12 exons, other *DEK* genes possessed 11 exons (Figure 3b). In addition, closely related genes had more similar arrangements of gene structure. There were 15 conserved motifs (motif 1 to 15) in DEKs were identified by MEME software. The group I DEK proteins had fourteen motifs (except motif 14), the group II DEK (GbDEK3D) protein had nine motifs (motif 1, 2, 3, 4, 5, 10, 11, 12 and 13), and the group III DEK proteins contained all motifs (Figure 3c), indicating that they are highly conserved among DEKs. We also identified domains in the DEKs using Batch Web CD-Search Tool, so as to gene structure, closely related proteins had more similar arrangements of motifs and domains (Figure 3d).

(a) Phylogenetic tree of *DEK* genes. The ML phylogenetic tree was constructed by using MEGA X with 1000 replicates. (b) Exon-intron organization analyses of *DEK* genes. Yellow boxes mean Untranslated Regions (UTR), Exons and introns are distinguished in green boxes and in black lines, respectively. (c) Conserved motifs of DEK proteins. Fifteen conserved motifs were identified through a MEME analysis, represented by different color boxes, and protein length was estimated via the scale at the bottom. (d) The conserved domains of DEK proteins.

### 3.4 Prediction of cis-acting elements in the promoters of GhDEKs

The 2.0 kb upstream promoter regions of *GhDEK*s were obtained and analyzed to predict the possible biological functions. Response to various stresses were our main focus, for instance salicylic acid, abscisic acid, defense, auxin, methyl jasmonate (MeJA), drought, etc., (Table 1). The abundance of cis-acting showed that *GhDEK* genes might perform different biological functions.

**Table 1.** Prediction of cis-acting regulatory elements about various responses of *GhDEK* genes

	SA	JA	ABA	Auxin	Defense and stress	Drought
GhDEK1A	0	2	2	2	1	0
GhDEK1D	0	2	2	2	1	0
GhDEK2A	1	4	0	0	0	2
GhDEK2D	0	6	4	0	0	0
GhDEK4A	1	2	2	0	2	1
GhDEK4D	2	2	3	0	2	0

### 3.5 Expression patterns of GhDEKs in tissues of cotton

**Figure 4.**
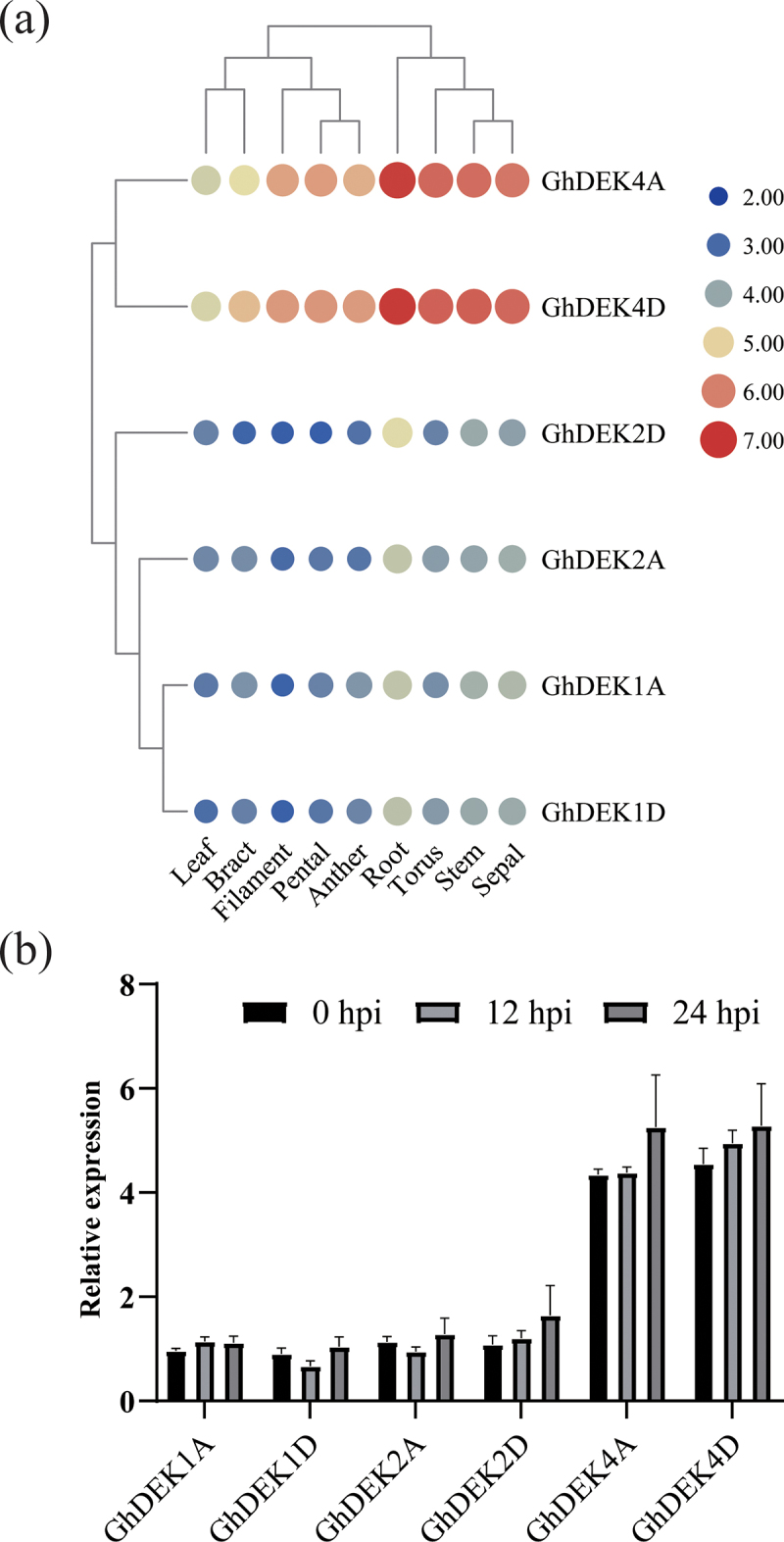
Expression pattern analysis of *GhDEK* genes in tissues and under stress.

To explore the functions of the *GhDEK*s, the expression patterns of the six *GhDEK*s were analyzed in leaf, bract, filament, pental, anther, root, torus, stem, and sepal. *GhDEK4A* and *GhDEK4D* were higher than other genes in all tissues. All the genes expression were the highest in root (Figure 4a). *Vd* is a soil-borne fungus, which infests cotton roots, the abundance of DEKs in roots portend that DEKs might play an important role in against soil-borne fungus.

(a) The expression patterns of *GhDEK* genes in tissues (leaf, bract, filament, pental, anther, root, torus, stem and sepal.) were exhibited from the TM-1 transcriptome data. (b) The expression of *GhDEK*s under Vd080 were exhibited through RT-qPCR.

### 3.6 Expression patterns and post-translational modifications of GhDEKs under V. dahliae challenge

In the Vw-resistant cotton (Zhongzhimian NO. 2) plants treated with Vd080, at 12 hpi and 24 hpi, *GhDEK*s expression had no significant change compared with 0 hpi (Figure 4b). Under *Vd* challenge, only GhDEK2D had been phosphorylated, acetylated and ubiquitylated. A day after inoculation, GhDEK2D was phosphorylated, deubiquitylated in cotton resistant cultivar. GhDEK2D acetylation was activated in susceptible cultivar one and two days after inoculation. The mass spectrometry results showed that phosphorylation sites were Thr327 and Ser329, acetylation and ubiquitylation sites were Lys273 (Fig. S1). The results suggested that the GhDEK2D was important in response to *Vd* infection.

### 3.7 GhDEK2D silencing reduced the resistance of cotton to Vd

**Figure 5.**
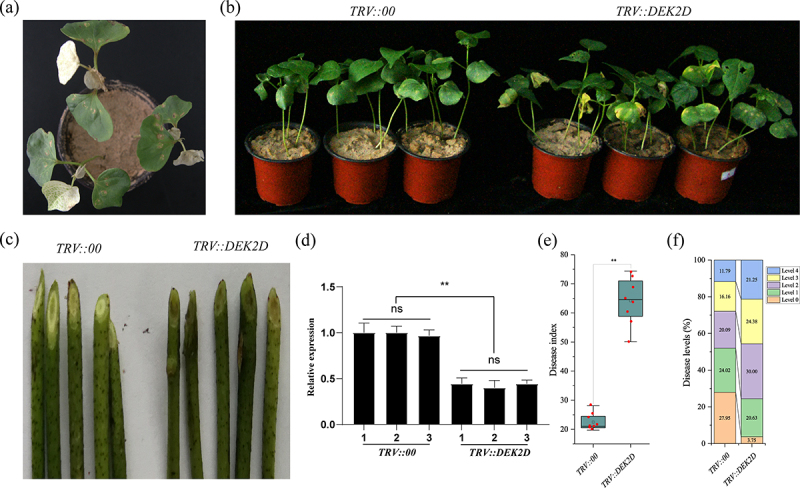
Knock-down of *GhDEK2D* attenuates plant resistance to *V. dahliae.*

To verify the function of *GhDEK2D* resistance to *Vd* in cotton, the transcript activity of *GhDEK2D* was restrained by VIGS. When Zhongzhimian NO. 2 seedlings had grown for 10 days, we infiltrated cotyledons with *TRV::00, TRV::GhDEK2D* or *TRV::PDS* (a visible gene marker). When the albino phenotype appeared in *TRV::GhPDS* infected newly true leaves (Figure 5a). After 1 week of treatment with Vd080 by root dipping method. Compared with the *TRV::00* plants, the morbidity of silencing plants was higher, such as wilted leaves and dark vascular bundles (Figure 5b,c). The disease index (Figure 5e) and disease levels (Figure 5f) were consistent with the plant phenotype. The results showed that the *GhDEK2D* silencing could attenuate plant resistance to *Vd* infection.

(a) Albino phenotype of *GhPDS* appeared 10 days after injection. (b) Disease phenotype of cotton plants post-infection with Vd080. Seedlings were inoculated with *Vd* a week after VIGS and photographed 30 days later. (c) Compared with the control plants, the silencing cottons were higher dark vascular bundles. The successful knock-down of the *GhDEK2D* transcript was confirmed by the qPCR analysis (d). The disease index (e) and disease levels analysis (f) of the plant were calculated. The data represent the mean ± SD, n = 3 (biological replicates) (*p < .05; **p < .01, *t* test).

### 3.8 GhDEK2D promotes plant defensive reaction

**Figure 6.**
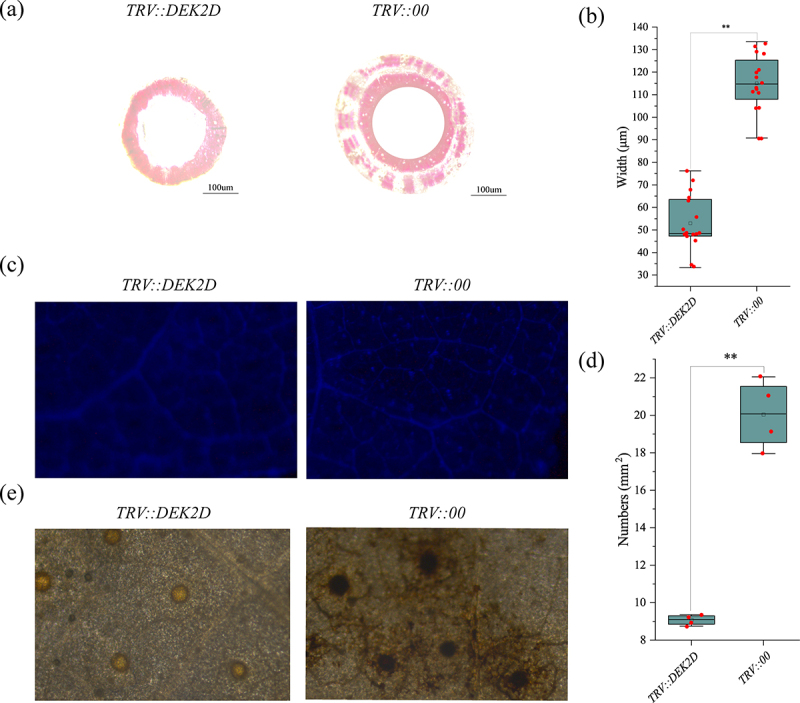
*GhDEK2D* promotes plant defensive reaction.

In order to explore the mechanism of *GhDEK2D* resistance to *Vd* infection, the cotton xylem was observed. A noticeable increase in the thickness of xylem in the control plants was observed (Figure 6a). The thickness of xylem were 52.99 μm and 115.09 μm in *TRV::GhDEK2D* and *TRV::00*, respectively (Figure 6b). A decreased density of callose depositions (number per mm^2^) was visualized in the leaves of silencing plants by staining with aniline blue (Figure 6c,d). The ROS changed in the control group was stronger (Figure 6e). Thesa results suggested that *GhDEK2D* silencing could reduce physical defense.

The phenotype (a) and thickness (b) of xylem in the cotton plants were observed. Scale bar, 100 μm. (c, d) Callose deposition in leaves of *TRV::00* and *TRV:GhDEK2D* cottons. Leaves were collected for aniline blue staining. Microscopy images were shown (c) and the number of callose deposits was recorded (d). Scale bar, 2 mm. (e) The ROS changed in the control group was stronger. The data represent the mean ± SD, n = 3 (biological replicates) (*p < .05; **p < .01, *t* test).

**Figure 7.**
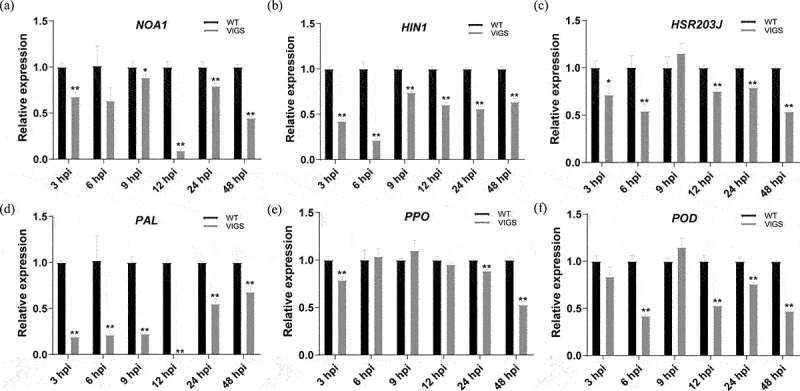
Expression analysis of defense-related genes in roots of *GhDEK2D*-silenced and control cotton plants.

The expressions of cotton defense-related genes were tested by RT-qPCR in root. *NOA* is a gene related to nitricoxide synthesis pathway, *NOA1* expression of *TRV::00* plants were significantly higher than *TRV::GhDEK2D* at 3, 9, 12, 24 and 48 hpi (Figure 7a) *HIN1* and *HSR203J* are HR marker gene. *HIN1* expression of *TRV::GhDEK2D* were inhibited all the time (Figure 7b). HSR203J expression of *TRV::GhDEK2D* were inhibited at 3, 6, 12, 24 and 48 hpi (Figure 7c). *PAL, PPO* and *POD* play important roles in plant immunology. In our study, *PAL* expression of *TRV::00* plants were significantly higher than *TRV::GhDEK2D* all the time (Figure 7d); *PPO* expression of *TRV::00* plants were significantly higher than *TRV::GhDEK2D* at 3, 24 and 48 hpi (Figure 7e). At 6, 12, 24 and 48 hpi, *POD* expression was significant increase in *TRV::00* plants, but it also had a quick low afterward (Figure 7f). The results suggested that the expressions of defense-related genes were broke in silenced cotton plants.

The expression levels of *GhNOA1* (a), *GhHIN1* (b), *GhHSR203J* (c), *GhPAL* (d), *GhPPO* (e) and *GhPOD* (f) defense-related genes under Vd080 inoculation in 3, 6, 9, 12, 24, and 48 hpi. The data represent the mean ± SD, n = 3 (biological replicates and technical replicates) (*p < .05; **p < .01, *t* test).

### 3.9 Overexpression of GhDEK2D enhances Arabidopsis resistance to Vd

**Figure 8.**
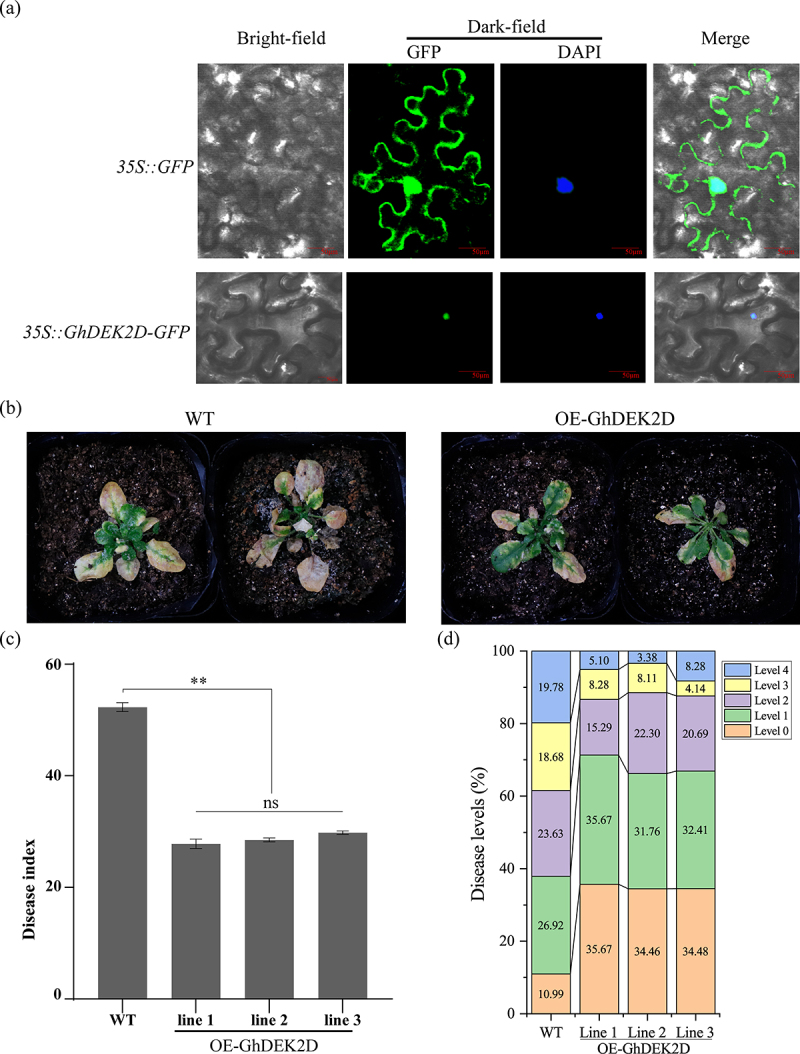
Enhanced disease resistance of *Arabidopsis* plants overexpressing GhDEK2D.

In order to analyze the subcellular localization of GhDEK2D, by heterologous expression in tobacco leaves, we observed GhDEK2D was located on the cell nucleus of tobacco cells (Figure 8a). The resistance of overexpression of GhDEK2D transgenic plants to *Vd* was assessed with an in vitro technique. GhDEK2D-overexpressing *Arabidopsis* were more resistant to Vw than the WT plants (Figure 8b,c). These results indicated that GhDEK2D overexpression enhances *Arabidopsis* resistance to *Vd*.

(a) The GhDEK2D was transiently expressed in tobacco leaves, GFP and DAPI staining were observed using confocal microscopy. GhDEK2D was located on cell nucleus of tobacco cells. DAPI is a cell nucleus-specific fluorescent probe. Scale bar, 50 μm. Symptoms of wild-type (WT), OE-GhDEK2D plants in nutrient soil at three weeks (b) after inoculation with *Vd*. The disease index (c) and disease levels (d) of WT and transgenic plants. Error bars indicate the SD of three biological replicates. The data represent the mean ± SD, n = 3 (biological replicates) (*p < .05; **p < .01, *t* test).

## 4 Disscussion

Chromatin plays a major role in the regulation of DNA-dependent processes. DEK as an architectural chromatin protein, which is associated with DNA, chromatin, and histone binding as well as DNA-folding activities.^51^ in *Arabidopsis thaliana*, there are four *DEK* genes, they are named *DEK1, DEK2, DEK3*, and *DEK4*, respectively.^52^ In *G. hirsutum*, six DEK proteins were named GhDEK1A, GhDEK1D, GhDEK2A, GhDEK2D, GhDEK4A, and GhDEK4D. The 60 DEK proteins were used for constructing a phylogenetic tree from 14 species. The same species proteins were clustered together, such as: SlDEKs, AtDEKs, LuDEKs, and so on (Figure 1). In cottons, gene structure, conserved motifs and domain of DEKs were semblable (Figure 3). The results suggested *DEK*s were conserved at the evolution.

Post-translationally can regulate a lot of plant receptors and critical signaling nodes, so that plant can rapidly and appropriately respond to biotic stresses. Protein is phosphorylated on serine, threonine, and tyrosine residues, which is a reversible reaction.^53^ Phosphorylation is major defense mechanism for controlling plant immune.^14^ The phosphorylation of WRKY33 upregulates camalexin biosynthesis in *Arabidopsis* upon pathogen infection.^54^ MPK3 and MPK6 phosphorylated ERF72 and improved its transactivation activity, resulting in increased camalexin concentration and increased resistance to *Botrytis cinerea*.^55^ The DEK (human chromatin protein) is phosphorylated by the protein kinase CK2.^56^ Acetylation of DEK affects the sub-nuclear localization and the ability to bind DNA.^57^ Fbxw7 acts as a tumor suppressor targeting multiple transcriptional activators and may target DEK for degradation to influence murine intestinal homeostasis and cancer by DEK ubiquitination.^58^ The intricate network of phosphorylation, acetylation, and ubiquitination mediated plant immunity had been reported. However, phosphorylation, acetylation, and ubiquitination of DEK hasn’t been studied. In this study, GhDEK2D had been phosphorylated, acetylated, and ubiquitylated under *Vd* challenge. As a positive regulator disease-resistant protein, GhDEK2D activated immunity by phosphorylated in cotton-resistant cultivar, and it was influenced by ubiquitylated in cotton-susceptible cultivar. GhDEK2D acetylation was activated in susceptible cultivar one and two days after inoculation, however, the function of DEK acetylation was unclear. The network of GhDEK2D phosphorylation, acetylation, and ubiquitination still needs further research.

In *GhDEK2D*-silenced cotton plants, the disease index was increscent (Figure 5), and the accumulation of ROS and callose in leaves, and xylogen in stems were reduced (Figure 6). ROS is an early events responses and a signal that activates downstream biochemical reaction.^59^ Deposition of callose at the cell wall is used in response to stress.^60^ Xylogen is a physical mechanism of plant stress resistance.^61^ Nitric oxide associated factor (NOA) is related to nitricoxide synthesis pathway, and indirectly regulates nitric oxide (NO) accumulation and response to stress.^62,63^ *HIN1* and *HSR203J* are another specific marker gene for HR cell death.^64,65^ In the current study, the expression of *GhNOA1, GhHIN1*, and *GhHSR203J* were inhibited in silenced cotton plants (Figure 7a,b,c). PAL, POD and PPO as defense enzymes to involve in the formation of barriers against pathogens,^66–68^ the expression of disease-resistant genes in *GhDEK2D* silenced cottons were significantly decreased after *Vd* inoculation (Figure 7d,e,f). These changes lead to *Vd* was more likely to infect silenced cotton.

In *Arabidopsis*, DEK plays a central role in balancing the response between growth and arrest.^69^ *Arabidopsis* DEK3 and DEK4 facilitate the transcription of key flowering repressors by interacting with chromatin, and prevent precocious flowering. Tomato DEK involves in defense response regulation to *Botrytis cinerea* and *Pseudomonas syringae*.^27^ Overexpression of GhDEK2D enhances *Arabidopsis* resistance to *Vd*. However, the molecular mechanism of GhDEK2D against pathogen, which we need do in the future.

## Supplementary Material

Supplemental Material
