# Supplementary material for: A DEK domain-containing protein GhDEK2D mediated Gossypium hirsutum enhanced resistance to Verticillium dahliae
Source: Plant Signal Behav. 2022 Jan 16;17(1):2024738. doi: 10.1080/15592324.2021.2024738 (PMC9176258; doi:10.1080/15592324.2021.2024738)
Supplement: Supplemental Material [file KPSB_A_2024738_SM3625.zip › Table S1.docx]

Table S1. Characteristics of *DEK* genes in cotton (*G. hirsutum*, *G. arboretum*, *G. raimondii* and *G. barbadense*)

| Gene ID | Gene Name | Chromosome | Start | End | Strand | CDS length (bp) | Protein  length  (a.a.) | MW | IP |
| --- | --- | --- | --- | --- | --- | --- | --- | --- | --- |
| Ga12G0910 | GaDEK1 | Chr12 | 9333414 | 9338178 | - | 1593 | 530 | 59.072 | 5.706 |
| Ga07G0580 | GaDEK2 | Chr07 | 6354602 | 6359318 | - | 1614 | 537 | 59.869 | 5.059 |
| Ga05G4239 | GaDEK4 | Chr05 | 96200930 | 96205683 | + | 1929 | 642 | 73.402 | 4.571 |
| Gorai.008G208200 | GrDEK1 | Chr08 | 49315157 | 49321082 | + | 1593 | 530 | 59.124 | 6.98 |
| Gorai.009G450100 | GrDEK4 | Chr09 | 70106118 | 70112139 | + | 1926 | 641 | 73.383 | 4.604 |
| Gbar_A12G020420 | GbDEK1A | A12 | 93411210 | 93416718 | + | 1596 | 531 | 59.214 | 5.557 |
| Gbar_D12G020610 | GbDEK1D | D12 | 51291396 | 51296993 | + | 1584 | 527 | 58.782 | 7.503 |
| Gbar_A07G005300 | GbDEK2A | A07 | 6266662 | 6272084 | - | 1617 | 538 | 59.769 | 5.02 |
| Gbar_D07G005580 | GbDEK2D | D07 | 5841856 | 5847244 | - | 1611 | 536 | 59.536 | 5.19 |
| Gbar_D01G008480 | GbDEK3D | D01 | 11839188 | 11843157 | + | 1083 | 360 | 41.333 | 6.58 |
| Gbar_A04G000620 | GbDEK4A | A04 | 834226 | 840466 | - | 1929 | 642 | 73.376 | 4.571 |
| Gbar_D05G039330 | GbDEK4D | D05 | 63255624 | 63261878 | + | 1926 | 641 | 73.373 | 4.604 |
| Gh_A12G214900 | GhDEK1A | A12 | 96631162 | 96635921 | + | 1593 | 530 | 59.1 | 5.557 |
| Gh_D12G208000 | GhDEK1D | D12 | 54121586 | 54127065 | + | 1590 | 529 | 59.076 | 8.033 |
| Gh_A07G053600 | GhDEK2A | A07 | 6330261 | 6335630 | - | 1593 | 530 | 58.827 | 5.238 |
| Gh_D07G054200 | GhDEK2D | D07 | 5779625 | 5784950 | - | 1602 | 533 | 59.229 | 5.139 |
| Gh_A04G006300 | GhDEK4A | A04 | 612553 | 618761 | - | 1929 | 642 | 73.261 | 4.591 |
| Gh_D05G399200 | GhDEK4D | D05 | 63548611 | 63554869 | + | 1923 | 640 | 73.243 | 4.625 |
