# Supplementary material for: A DEK domain-containing protein GhDEK2D mediated Gossypium hirsutum enhanced resistance to Verticillium dahliae
Source: Plant Signal Behav. 2022 Jan 16;17(1):2024738. doi: 10.1080/15592324.2021.2024738 (PMC9176258; doi:10.1080/15592324.2021.2024738)
Supplement: Supplemental Material [file KPSB_A_2024738_SM3625.zip › Table S2.docx]

Table S2. The name of different species DEK proteins

| Gene ID | Name |
| --- | --- |
| At3g48710 | AtDEK1 |
| At5g63550 | AtDEK2 |
| At4g26630 | AtDEK3 |
| At5g55660 | AtDEK4 |
| Ga12G0910.1 | GaDEK1 |
| Ga07G0580.1 | GaDEK2 |
| Ga05G4239.1 | GaDEK4 |
| Gbar_A12G020420.1 | GbDEK1A |
| Gbar_D12G020610.1 | GbDEK1D |
| Gbar_A07G005300.1 | GbDEK2A |
| Gbar_D07G005580.1 | GbDEK2D |
| Gbar_D01G008480.1 | GbDEK3D |
| Gbar_A04G000620.1 | GbDEK4A |
| Gbar_D05G039330.1 | GbDEK4D |
| Godar.A12G219300 | GdDEK1A |
| Godar.D12G227200 | GdDEK1D |
| Godar.A07G058000 | GdDEK2A |
| Godar.D07G059000 | GdDEK2D |
| Godar.A04G008600 | GdDEK4A |
| Godar.D05G454100 | GdDEK4D |
| Gh_A12G214900.1 | GhDEK1A |
| Gh_D12G208000.1 | GhDEK1D |
| Gh_A07G053600.1 | GhDEK2A |
| Gh_D07G054200.1 | GhDEK2D |
| Gh_A04G006300.1 | GhDEK4A |
| Gh_D05G399200.1 | GhDEK4D |
| Gomus.A12G206700 | GmDEK1A |
| Gomus.D12G218200 | GmDEK1D |
| Gomus.A07G055800 | GmDEK2A |
| Gomus.D07G057600 | GmDEK2D |
| Gomus.A04G007000 | GmDEK4A |
| Gorai.008G208200.1 | GrDEK1 |
| Gorai.009G450100.1 | GrDEK4 |
| Gotom.A12G219900 | GtDEK1A |
| Gotom.D12G227900 | GtDEK1D |
| Gotom.A07G057300 | GtDEK2A |
| Gotom.D07G059000 | GtDEK2D |
| Gotom.A04G007900 | GtDEK4A |
| Gotom.D05G448000 | GtDEK4D |
| Lus10035905 | LsDEK1 |
| Lus10016746 | LsDEK2 |
| Lus10025759 | LsDEK3 |
| Lus10022439 | LsDEK4 |
| Manes.14G048600 | MeDEK1 |
| Manes.06G122400 | MeDEK2 |
| Manes.03G140900 | MeDEK3 |
| Manes.15G060500 | MeDEK4 |
| Potri.015G100700 | PtDEK2 |
| Potri.011G093200 | PtDEK3 |
| Potri.001G367400 | PtDEK4 |
| Soltu.DM.06G027990 | StDEK1 |
| Soltu.DM.03G020170 | StDEK2 |
| Soltu.DM.10G001140 | StDEK3 |
| Soltu.DM.07G027230 | StDEK4 |
| Solyc07g065490 | SyDEK3 |
| Solyc10g006000 | SyDEK4 |
| ZmB84.03G193900 | ZmDEK1 |
| ZmB84.08G247500 | ZmDEK2 |
| ZmB84.05G072400 | ZmDEK3 |
| ZmB84.01G390800 | ZmDEK4 |
