# Supplementary material for: A DEK domain-containing protein GhDEK2D mediated Gossypium hirsutum enhanced resistance to Verticillium dahliae
Source: Plant Signal Behav. 2022 Jan 16;17(1):2024738. doi: 10.1080/15592324.2021.2024738 (PMC9176258; doi:10.1080/15592324.2021.2024738)
Supplement: Supplemental Material [file KPSB_A_2024738_SM3625.zip › Table S3.docx]

Table S3. Information of the homologous genes and the gene type of *DEK* genes in *G. hirsutum*, *G. arboretum*, *G. raimondii* and *G. barbadense*

| **Genes of *G. hirsutum*** | |  | **Orthologous genes in *G. raimondii*** | |
| --- | --- | --- | --- | --- |
| ID | Name |  | Name | ID |
| Gh_A12G214900 | GhDEK1A |  | GrDEK1 | Gorai.008G208200.1 |
| Gh_D12G208000 | GhDEK1D |  | GrDEK1 | Gorai.008G208200.1 |
| Gh_A07G053600 | GhDEK2A |  | GrDEK1 | Gorai.008G208200.1 |
| Gh_D07G054200 | GhDEK2D |  | GrDEK1 | Gorai.008G208200.1 |
| Gh_A04G006300 | GhDEK4A |  | GrDEK4 | Gorai.009G450100.1 |
| Gh_D05G399200 | GhDEK4D |  | GrDEK4 | Gorai.009G450100.1 |
|  |  |  |  |  |
| **Genes of *G. hirsutum*** | |  | **Orthologous genes in *G. barbadense*** | |
| ID | Name |  | Name | ID |
| Gh_A12G214900 | GhDEK1A |  | GbDEK2A | Gbar_A07G005300 |
| Gh_A12G214900 | GhDEK1A |  | GbDEK1A | Gbar_A12G020420 |
| Gh_A12G214900 | GhDEK1A |  | GbDEK2D | Gbar_D07G005580 |
| Gh_D12G208000 | GhDEK1D |  | GbDEK2A | Gbar_A07G005300 |
| Gh_D12G208000 | GhDEK1D |  | GbDEK1A | Gbar_A12G020420 |
| Gh_D12G208000 | GhDEK1D |  | GbDEK2D | Gbar_D07G005580 |
| Gh_A07G053600 | GhDEK2A |  | GbDEK2A | Gbar_A07G005300 |
| Gh_A07G053600 | GhDEK2A |  | GbDEK1A | Gbar_A12G020420 |
| Gh_A07G053600 | GhDEK2A |  | GbDEK2D | Gbar_D07G005580 |
| Gh_D07G054200 | GhDEK2D |  | GbDEK2A | Gbar_A07G005300 |
| Gh_D07G054200 | GhDEK2D |  | GbDEK1A | Gbar_A12G020420 |
| Gh_D07G054200 | GhDEK2D |  | GbDEK2D | Gbar_D07G005580 |
| Gh_A04G006300 | GhDEK4A |  | GbDEK3D | Gbar_D01G008480 |
| Gh_A04G006300 | GhDEK4A |  | GbDEK4D | Gbar_D05G039330 |
| Gh_D05G399200 | GhDEK4D |  | GbDEK3D | Gbar_D01G008480 |
| Gh_D05G399200 | GhDEK4D |  | GbDEK4D | Gbar_D05G039330 |
|  |  |  |  |  |
| **Genes of *G. hirsutum*** | |  | **Orthologous genes in *G. arboretum*** | |
| ID | Name |  | Name | ID |
| Gh_A12G214900 | GhDEK1A |  | GaDEK2 | Ga07G0580 |
| Gh_A12G214900 | GhDEK1A |  | GaDEK1 | Ga12G0910 |
| Gh_D12G208000 | GhDEK1D |  | GaDEK2 | Ga07G0580 |
| Gh_D12G208000 | GhDEK1D |  | GaDEK1 | Ga12G0910 |
| Gh_A07G053600 | GhDEK2A |  | GaDEK2 | Ga07G0580 |
| Gh_A07G053600 | GhDEK2A |  | GaDEK1 | Ga12G0910 |
| Gh_D07G054200 | GhDEK2D |  | GaDEK2 | Ga07G0580 |
| Gh_D07G054200 | GhDEK2D |  | GaDEK1 | Ga12G0910 |
| Gh_A04G006300 | GhDEK4A |  | GaDEK4 | Ga05G4239 |
| Gh_D05G399200 | GhDEK4D |  | GaDEK4 | Ga05G4239 |
|  |  |  |  |  |
| **Genes of *G. barbadense*** | |  | **Orthologous genes in *G. raimondii*** | |
| ID | Name |  | Name | ID |
| Gbar_A12G020420 | GbDEK1A |  | GrDEK1 | Gorai.008G208200.1 |
| Gbar_A07G005300 | GbDEK2A |  | GrDEK1 | Gorai.008G208200.1 |
| Gbar_D07G005580 | GbDEK2D |  | GrDEK1 | Gorai.008G208200.1 |
| Gbar_D01G008480 | GbDEK3D |  | GrDEK4 | Gorai.009G450100.1 |
| Gbar_D05G039330 | GbDEK4D |  | GrDEK4 | Gorai.009G450100.1 |
|  |  |  |  |  |
| **Genes of *G. barbadense*** | |  | **Orthologous genes in *G. arboretum*** | |
| ID | Name |  | Name | ID |
| Gbar_A12G020420 | GbDEK1A |  | GaDEK2 | Ga07G0580 |
| Gbar_A12G020420 | GbDEK1A |  | GaDEK1 | Ga12G0910 |
| Gbar_A07G005300 | GbDEK2A |  | GaDEK2 | Ga07G0580 |
| Gbar_A07G005300 | GbDEK2A |  | GaDEK1 | Ga12G0910 |
| Gbar_D07G005580 | GbDEK2D |  | GaDEK2 | Ga07G0580 |
| Gbar_D07G005580 | GbDEK2D |  | GaDEK1 | Ga12G0910 |
| Gbar_D01G008480 | GbDEK3D |  | GaDEK4 | Ga05G4239 |
| Gbar_D05G039330 | GbDEK4D |  | GaDEK4 | Ga05G4239 |
|  |  |  |  |  |
| **Genes of *G. raimondii*** | |  | **Orthologous genes in *G. arboretum*** | |
| ID | Name |  | Name | ID |
| Gorai.008G208200.1 | GrDEK1 |  | GaDEK2 | Ga07G0580 |
| Gorai.008G208200.1 | GrDEK1 |  | GaDEK1 | Ga12G0910 |
| Gorai.009G450100.1 | GrDEK4 |  | GaDEK4 | Ga05G4239 |
|  |  |  |  |  |
| **Genes of *G. hirsutum*** | |  | **Paralogous genes in *G. hirsutum*** | |
| ID | Name |  | Name | ID |
| Gh_A12G214900 | GhDEK1A |  | GhDEK2D | Gh_D07G054200 |
| Gh_A12G214900 | GhDEK1A |  | GhDEK1D | Gh_D12G208000 |
| Gh_A07G053600 | GhDEK2A |  | GhDEK1A | Gh_A12G214900 |
| Gh_A07G053600 | GhDEK2A |  | GhDEK2D | Gh_D07G054200 |
| Gh_A07G053600 | GhDEK2A |  | GhDEK1D | Gh_D12G208000 |
| Gh_D07G054200 | GhDEK2D |  | GhDEK1D | Gh_D12G208000 |
| Gh_A04G006300 | GhDEK4A |  | GhDEK4D | Gh_D05G399200 |
|  |  |  |  |  |
| **Genes of *G. arboretum*** | |  | **Paralogous genes in *G. arboretum*** | |
| ID | Name |  | Name | ID |
| Ga07G0580 | GaDEK2 |  | GaDEK1 | Ga12G0910 |
|  |  |  |  |  |
| **Genes of *G. barbadense*** | |  | **Paralogous genes in *G. barbadense*** | |
| ID | Name |  | Name | ID |
| Gbar_A12G020420 | GbDEK1A |  | GbDEK2D | Gbar_D07G005580 |
| Gbar_A12G020420 | GbDEK1A |  | GbDEK1D | Gbar_D12G020610 |
| Gbar_A07G005300 | GbDEK2A |  | GbDEK1A | Gbar_A12G020420 |
| Gbar_A07G005300 | GbDEK2A |  | GbDEK2D | Gbar_D07G005580 |
| Gbar_A07G005300 | GbDEK2A |  | GbDEK1D | Gbar_D12G020610 |
| Gbar_D07G005580 | GbDEK2D |  | GbDEK1D | Gbar_D12G020610 |
| Gbar_D01G008480 | GbDEK3D |  | GbDEK4D | Gbar_D05G039330 |
| Gbar_A04G000620 | GbDEK4A |  | GbDEK3D | Gbar_D01G008480 |
| Gbar_A04G000620 | GbDEK4A |  | GbDEK4D | Gbar_D05G039330 |
|  |  |  |  |  |
| **Genes of *G. raimondii*** | |  | **Paralogous genes in *G. raimondii*** | |
| ID | Name |  | Name | ID |
|  |  |  |  |  |
| **Genes of *G. hirsutum*** | | | |  |
| ID | Name | Genetype | |  |
| Gh_A12G214900.1 | GhDEK1A | WGD or Segmental | |  |
| Gh_D12G208000.1 | GhDEK1D | WGD or Segmental | |  |
| Gh_A07G053600.1 | GhDEK2A | WGD or Segmental | |  |
| Gh_D07G054200.1 | GhDEK2D | WGD or Segmental | |  |
| Gh_A04G006300.1 | GhDEK4A | WGD or Segmental | |  |
| Gh_D05G399200.1 | GhDEK4D | WGD or Segmental | |  |
|  |  |  |  |  |
| **Genes of *G. barbadense*** | | | |  |
| ID | Name | Genetype | |  |
| Gbar_A12G020420.1 | GbDEK1A | WGD or Segmental | |  |
| Gbar_D12G020610.1 | GbDEK1D | WGD or Segmental | |  |
| Gbar_A07G005300.1 | GbDEK2A | WGD or Segmental | |  |
| Gbar_D07G005580.1 | GbDEK2D | WGD or Segmental | |  |
| Gbar_D01G008480.1 | GbDEK3D | WGD or Segmental | |  |
| Gbar_A04G000620.1 | GbDEK4A | WGD or Segmental | |  |
| Gbar_D05G039330.1 | GbDEK4D | WGD or Segmental | |  |
|  |  |  |  |  |
| **Genes of *G. arboretum*** | | | |  |
| ID | Name | Genetype | |  |
| Ga12G0910.1 | GaDEK1 | WGD or Segmental | |  |
| Ga07G0580.1 | GaDEK2 | WGD or Segmental | |  |
| Ga05G4239.1 | GaDEK4 | WGD or Segmental | |  |
|  |  |  |  |  |
| **Genes of *G. raimondii*** | | | |  |
| ID | Name | Genetype | |  |
| Gorai.008G208200.1 | GrDEK1 | WGD or Segmental | |  |
| Gorai.009G450100.1 | GrDEK4 | WGD or Segmental | |  |
