# Supplementary material for: A DEK domain-containing protein GhDEK2D mediated Gossypium hirsutum enhanced resistance to Verticillium dahliae
Source: Plant Signal Behav. 2022 Jan 16;17(1):2024738. doi: 10.1080/15592324.2021.2024738 (PMC9176258; doi:10.1080/15592324.2021.2024738)
Supplement: Supplemental Material [file KPSB_A_2024738_SM3625.zip › Table S4.docx]

**S1 Table. Primers used in this stud**

| Gene name |  | Primer sequence (5'-3') | Purposes |
| --- | --- | --- | --- |
| *qPCR-GhDEK1A* | F: | GAGAAAGTGGATGAGATGAAAGA | RT-qPCR for *GhDEK1A* |
|  | R: | TTTTTTGTCTTCCCCTTAACTT |  |
| *qPCR-GhDEK1D* | F: | AAGGTGGATGAGTTGAAAGAA | RT-qPCR for *GhDEK1D* |
|  | R: | CTTCTTTGTCTTCCCCTTAAC |  |
| *qPCR-GhDEK2A* | F: | GGAGGAAGAGGAAGAAGAAGA | RT-qPCR for *GhDEK2A* |
|  | R: | CAACAGAAGGAGCTGAAT |  |
| *qPCR-GhDEK2D* | F: | ACCTGAGCCTAGTAGGGAAG | RT-qPCR for *GhDEK2D* |
|  | R: | GATGAGGATGAGGAAGGGGA |  |
| *qPCR-GhDEK4A* | F: | AGCCACAGGTAAAGGCAAAAG | RT-qPCR for *GhDEK4A* |
|  | R: | TCCCATTCCCATCTTTAT |  |
| *qPCR-GhDEK4D* | F: | GAGCAGGAAAAACAAAAGGC | RT-qPCR for *GhDEK4D* |
|  | R: | TCCCATTCCCATCTTTAT |  |
| *VIGS-GhDEK2D* | F: | GCTCTAGAAGGCTATGTTTGGGTT | VIGS for *GhDEK2D* |
|  | R: | GGGGTACCTTTACCCTTCTGTTCC |  |
| *OE-GhDEK2D* | F: | CACGGGGGACTCTAGAATGGCGACGGAAACCCTAG | Vector construction for p*CAMBIA3300-eGFP* |
|  | R: | TGTTAATTAAGGATCCGGCATCATCATCTCCATCACC |  |
| *GhNOA1* | F: | GAGGATGCTGAAAGACCTGCTA | RT-qPCR for *GhNOA1* |
|  | R: | TCTCAACTGGCTTGGGTACATG |  |
| *GhHIN1* | F: | GCTGATGAGACATCGGAGTTTA | RT-qPCR for *GhHIN1* |
|  | R: | CTACCATTCCCAGTGTTCAAAG |  |
| *GhHSR203J* | F: | GTGATAGCTCAGGAGGGAACAT | RT-qPCR for *GhHSR203J* |
|  | R: | CTAACTCGGACTTGCTTCGTTG |  |
| *GhPAL* | F: | TGGTGGCTGAGTTTAGGAAA | RT-qPCR for *GhPAL* |
|  | R: | TGAGTGAGGCAATGTGTGA |  |
| *GhPOD* | F: | ATATCCTTGTTCTGTCTGCTA | RT-qPCR for *GhPOD* |
|  | R: | CTCCTTCTACCGTCTCTTC |  |
| *GhPPO* | F: | ATATCCTTGTTCTGTCTGCTA | RT-qPCR for *GhPPO* |
|  | R: | CTCCTTCTACCGTCTCTTC |  |
| *GhUBQ7* | F: | GAGTCTTCGGACACCATTG | RT-qPCR for endogenous reference gene of *Gossypium hirsutum* |
|  | R: | CTTGACCTTCTTCTTCTTGTGC |  |
| *AtUBQ10* | F: | GTATGGCTTCTCGTTCACAT | RT-qPCR or qPCR for endogenous reference gene of *Arabidopsis thaliana* |
|  | R: | CTAAGAGGCAACTGCAGACT |  |
| *35S::DEK2D* | F: | TTGCGATAAAGGAAAGGC | PCR for GhDEK2D in *Arabidopsis thaliana* |
|  | R: | CATTCTCAACCCAAACATA |  |
